# Spindle pole body component 25 and platelet-derived growth factor mediate crosstalk between tumor-associated macrophages and prostate cancer cells

**DOI:** 10.3389/fimmu.2022.907636

**Published:** 2022-07-27

**Authors:** Feilun Cui, Zhipeng Xu, Jianpeng Hu, Yumei Lv

**Affiliations:** ^1^ Department of Urology, Affiliated People’s Hospital of Jiangsu University, Zhenjiang, China; ^2^ Department of Health Management Section, Zhenjiang College, Zhenjiang, China

**Keywords:** SPC25, tumor associated macrophage (TAM), prostate cancer, pdgf, crosstalk

## Abstract

Tumor-associated macrophages (TAMs) are involved in the growth of prostate cancer (PrC), while the molecular mechanisms underlying the interactive crosstalk between TAM and PrC cells remain largely unknown. Platelet-derived growth factor (PDGF) is known to promote mesenchymal stromal cell chemotaxis to the tumor microenvironment. Recently, activation of spindle pole body component 25 (SPC25) has been shown to promote PrC cell proliferation and is associated with PrC stemness. Here, the relationship between SPC25 and PDGF in the crosstalk between TAM and PrC was investigated. Significant increases in both PDGF and SPC25 levels were detected in PrC specimens compared to paired adjacent normal prostate tissues. A significant correlation was detected between PDGF and SPC25 levels in PrC specimens and cell lines. SPC25 increased PDGF production and tumor cell growth in cultured PrC cells and in xenotransplantation. Mechanistically, SPC25 appeared to activate PDGF in PrC likely through Early Growth Response 1 (Egr1), while the secreted PDGF signaled to TAM through PDGFR on macrophages and polarized macrophages, which, in turn, induced the growth of PrC cells likely through their production and secretion of transforming growth factor β1 (TGFβ1). Thus, our data suggest that SPC25 triggers the crosstalk between TAM and PrC cells *via* SPC25/PDGF/PDGFR/TGFβ1 receptor signaling to enhance PrC growth.

## Introduction

Prostate cancer (PrC) has the highest incidence among malignancies in aged Chinese male patients (1). The growth of PrCs is regulated by androgen (2). On the other hand, PrC cells may proliferate independently on androgen (3) and metastasize to distant organs. Such cases are castration-resistant prostate cancers (CRPCs) (2).

Kinetochores are complexes with over 100 proteins to connect chromosomal DNA with spindle microtubules, and are essential for accurate chromosome segregation, whose dysfunction will lead to chromosome instability and tumorigenesis (4). Kinetochore–microtubule structure forms in metaphase for chromosomes to align in the middle of spindle, in which process the NDC80 complex is indispensable (4). The NDC80 complex is composed of four components: NDC80, NUF2, spindle pole body component 24 (SPC24), and SPC25. SPC25 is upregulated in lung adenocarcinoma, and plays roles in carcinogenesis, cancer cell proliferation, and metastasis (5). In addition, SPC25 is enriched in cancer stem cells (CSCs) (6), and is required for PrC cell proliferation (7).

Platelet-derived growth factor (PDGF) regulates the growth of many tissues and organs (8). PDGF ligands have several isoforms, from which alpha and beta are the most important PDGF receptor subunits. PDGF signaling plays important roles in the regulation of mesenchymal cell proliferation, differentiation, and migration (8). PDGF signaling is also associated with tumorigenesis (9), and is involved in the functionality of macrophages (10–12). However, it is noteworthy that a regulatory relationship between SPC25 and PDGF has not been reported before.

The first role of macrophages is phagocytosis of dying or dead cells, foreign substances, and microorganisms (13–16). These macrophages are termed M1. Macrophages can actually do much more than phagocytosis, such as participation in the control of inflammation, tissue repair, and regeneration (13–16). These macrophages are termed M2. Highly expressed nitric oxide synthase (iNOS) and high level of reactive oxygen species (ROS) are detected in M1 macrophages. M2 macrophage markers include CD206, CD163, arginase 1, and CD301. In addition, a group of macrophages detected in the tumor microenvironment is named tumor-associated macrophages (TAMs) (17), which share many features with M2 macrophages and promote tumorigenesis.

It was recently reported that M2 macrophages were infiltrated in the injured pancreas to secrete transforming growth factor β1 (TGFβ1) (18). Moreover, transplanting mesenchymal stem cells to the injured heart recruited macrophages, which accelerated the regeneration of cardiac muscles (19). However, the relation between PrC cells and TAM, especially with the involvement of SPC25 and PDGF, has not been investigated before. Thus, the above questions were addressed here.

## Methods

### Protocol approval

#### Ethics approval and consent or publication

PrC and paired adjacent normal prostate tissue were collected from 40 patients who had been treated and followed up in the Affiliated People’s Hospital of Jiangsu University. PrC patients were notified of research purposes and procedures, and written approvals were obtained from all patients. Human and animal protocols were approved by the institutional review board in the Affiliated People’s Hospital of Jiangsu University. Nude mice were used to minimize potential confounders. There was no exclusion of animals and data, and no humane endpoints in this research.

### PrC cell lines and transduction

Human PrC cell lines (LNCap, PC-3, BPH-1, and DU145), all originally from the American Type Culture Collection (ATCC, Rockville, MD, USA), were cultured in DMEM (Invitrogen, Shanghai, China) with 10% fetal bovine serum (FBS, Sigma-Aldrich, Beijing, China) at 37°C with 5% CO_2_. Cells were incubated with lentivirus (Applied Viromics, Fremont, CA, USA) with a plenti-CMV-LUC-2A-GFP vector (Clontech, Mountain View, CA, USA) at a multiplicity of infection (MOI) of 80 for 12 h. The transduced cells were isolated based on GFP by flow cytometry and were monitored *in vivo* based on luciferase expression in a luciferin assay.

### Tumor mouse model and bioluminescent surveillance of tumor

We transplanted 10^7^ AAV-transduced/labeled DU145 cells subcutaneously into 10-week-old male nude mice (SLAC Laboratory Animal Co. Ltd, Shanghai, China). In the next 56 days, an IVIS imaging system (Xenogen Corp., Alameda, CA, USA) was used to examine bioluminescence to assess tumor growth *in vivo*. Luciferin (150 mg/kg, Sigma-Aldrich) was intraperitoneally injected before taking mouse images and a bioluminescent quantification with Living Image software (Xenogen Corp.).

### Digesting tumor and characterizing dissociated cells by flow cytometry

Tumors by implanted PrCs were cut out and chopped, and then resuspended and digested with 40 mg/dl collagenase (Sigma-Aldrich) and 0.1% trypsin (Sigma-Aldrich) for 30 min at 37°C. Single cell suspension was incubated with conjugated antibodies against CD31, F4/80, and CD206 (Becton-Dickinson Biosciences, San Jose, CA, USA) before flow cytometric analysis. Data were analyzed by FlowJo software (Flowjo LLC, Ashland, OR, USA).

### Isolation of bone marrow-derived macrophages

Firstly, we isolated bones from male C57BL/6 mice at 12 weeks old and used a 26-gauge needle to flush out cells with macrophage-specific culture media. The collected bone marrow was centrifuged at 1,200 RPM for 5 min at 4°C. We re-suspended the pellets and plated them in a 24-well plate at a density of 10^6^ cells/well for culturing and new media was replaced every 2 days.

### Transwell co-culture system

DU145 cells (1 × 10^5^) and bone-marrow-derived macrophages (1 × 10^5^) were co-cultured in the system with/without SB431542 (10 µmol/L, Abcam, Cambridge, MA, USA), or with/without recombinant PDGF (150 ng/ml, Sigma-Aldrich, St. Louis, MO, USA), or with/without anti-PDGFR (15 µg/L, R&D Biosystem, Los Angeles, CA, USA) for 2 days. After co-culture, an MTT assay was applied to detect the number of DU145 cells and recorded the number variation. In addition, flow cytometry was applied to assess macrophage subtypes.

### MTT assay and determination of DNA content

The experiment was performed according to the brochure of the manufacturer, for observation and evaluation of the condition of growing cells by an MTT Viability assay Kit (Roche, Indianapolis, IN, USA). The next step was extraction of DNA, which was accomplished by a DNA isolation kit (Qiagen, Hilden, Germany). Finally, we used a Nanotrop 2000 machine (Thermo Scientific, Rockford, IL, USA) to assess the content of DNA.

### RT-qPCR

Total RNA was extracted from cells with RNeasy mini kit (Qiagen, Shanghai, China). Two micrograms of total RNA was used to reversely transcribe cDNA with Omniscript RT kit (Qiagen, Hilden, Germany). qPCR was performed with a QuantiTect SYBR Green PCR kit (Qiagen). Commercial primers were all bought from Qiagen and data were analyzed by the 2^-△△Ct^ method. We normalized mRNA expression of genes of interest to a housekeeping gene, GAPDH, and experimental controls.

### Protein analysis by ELISA, Western blot, and immunocytochemistry

Total cellular protein was extracted using RIPA buffer (Sigma-Aldrich) and quantified with BCA assay (Sigma-Aldrich). SPC25 and PDGF were measured with ELISA kits from MyBiosource and R&D Biosystem, respectively. Western blot was performed with the following antibodies: a rabbit anti-human SPC25 (1:750; Ab236972, Abcam, Dallas, TX, USA), a mouse anti-human Egr-1 (1:2,000; Ab55160, Abcam), a rabbit anti-human PDGF (1:1,000; Ab23914, Abcam), and a mouse anti-human GAPDH antibody (1:1,000; Ab8245, Abcam). All secondary antibodies were from Jackson ImmunoResearch Labs (West Grove, PA, USA). Quantification was done with ImageJ (NIH, Bethesda, MA, USA). Immunocytochemistry was done with a mouse anti-human PDGFR alpha antibody (1:50; Ab96569, Abcam).

### Statistics

Statistical analysis was done with GraphPad Prism (La Jolla, CA, USA). Difference between two groups was analyzed with unpaired two-tailed Student’s *t*-test, and difference between three or more groups was compared by one-way ANOVA with the Tukey posttest. Spearman’s rank correlation coefficient was calculated. Data were shown as mean ± SD, and *p* < 0.05 was considered statistically significant.

## Results

### SPC25 increases PDGF levels in PrC

**Figure 1:**
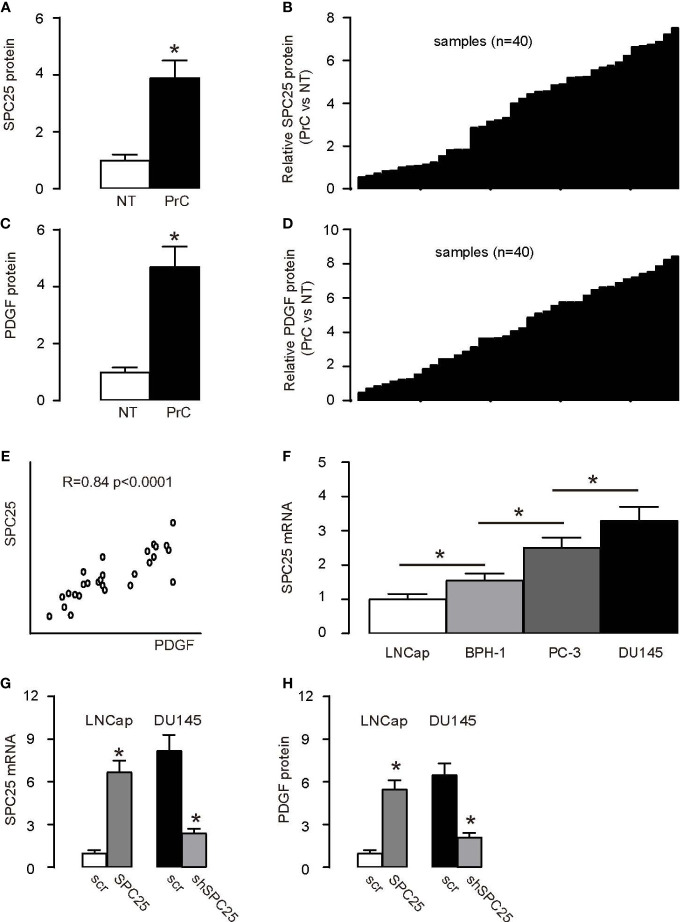
SPC25 increases PDGF levels in PrC **(A, B)** ELISA measurement of SPC25 protein level in 40 pairs of prostate cancer (PrC) and normal tissues (NT) as a group **(A)**, and as per pair **(B)**. **(C, D)** PDGF protein levels measured by ELISA in 40 pairs of PrC and NT, as a group **(A)**, and as per pair **(B)**. **(E)** Correlation between SPC25 and PDGF in PrC specimens (*R* = 0.84; *p* < 0.0001). **(F)** RT-qPCR for SPC25 in several human PrC cell lines. **(G)** mRNA level of SPC25 in human PrC cell lines DU145 and LNCap transfected by either shSPC25 or SPC25, compared to cells transfected by scrambled plasmids (scr). **(H)** ELISA for SPC25 in DU145 and LNCap transfected by either shSPC25 or SPC25, compared to cells transfected by scrambled plasmids (scr). *p < 0.05. *n* = 40 for clinical studies **(A–E)** and *n* = 5 for cell line studies **(F, G)**. Relative values were shown. For **(F, G)**, Scr = 1.

SPC25 protein expression measured by ELISA was significantly higher in PrC compared to paired normal prostate tissues (NT; *n* = 40) presented as a group (**Figure 1A**) and as each pair (**Figure 1B**). PrC samples and NT were then assessed for PDGF beta (from now on simplified as PDGF throughout this article) protein levels by ELISA. PrC specimens expressed significantly higher PDGF than NT shown by mean ± SD (**Figure 1C**) and by individual values (**Figure 1D**). Interestingly, a positive correlation between SPC25 and PDGF was detected in PrC specimens (**Figure 1E**). Next, we used several human PrC cell lines (LNCap, PC-3, BPH-1, and DU145) to verify the regulation of PDGF by SPC25. DU145 is androgen-insensitive without an androgen receptor and had the highest level of SPC25 among the above four cell lines, while LNCap is androgen-sensitive and expressed the lowest level of SPC25 (**Figure 1F**). Thus, LNCap cells were overexpressed with SPC25, and DU145 cells were transfected with shSPC25 so that SPC25 levels were significantly altered (**Figure 1G**). Interestingly, increases in SPC25 in LNCap cells significantly increased PDGF levels, while reduction in SPC25 in DU145 cells significantly decreased PDGF levels (**Figure 1H**). Thus, these data suggest that SPC25 may induce PDGF expression in PrC.

### PrC mouse model

**Figure 2:**
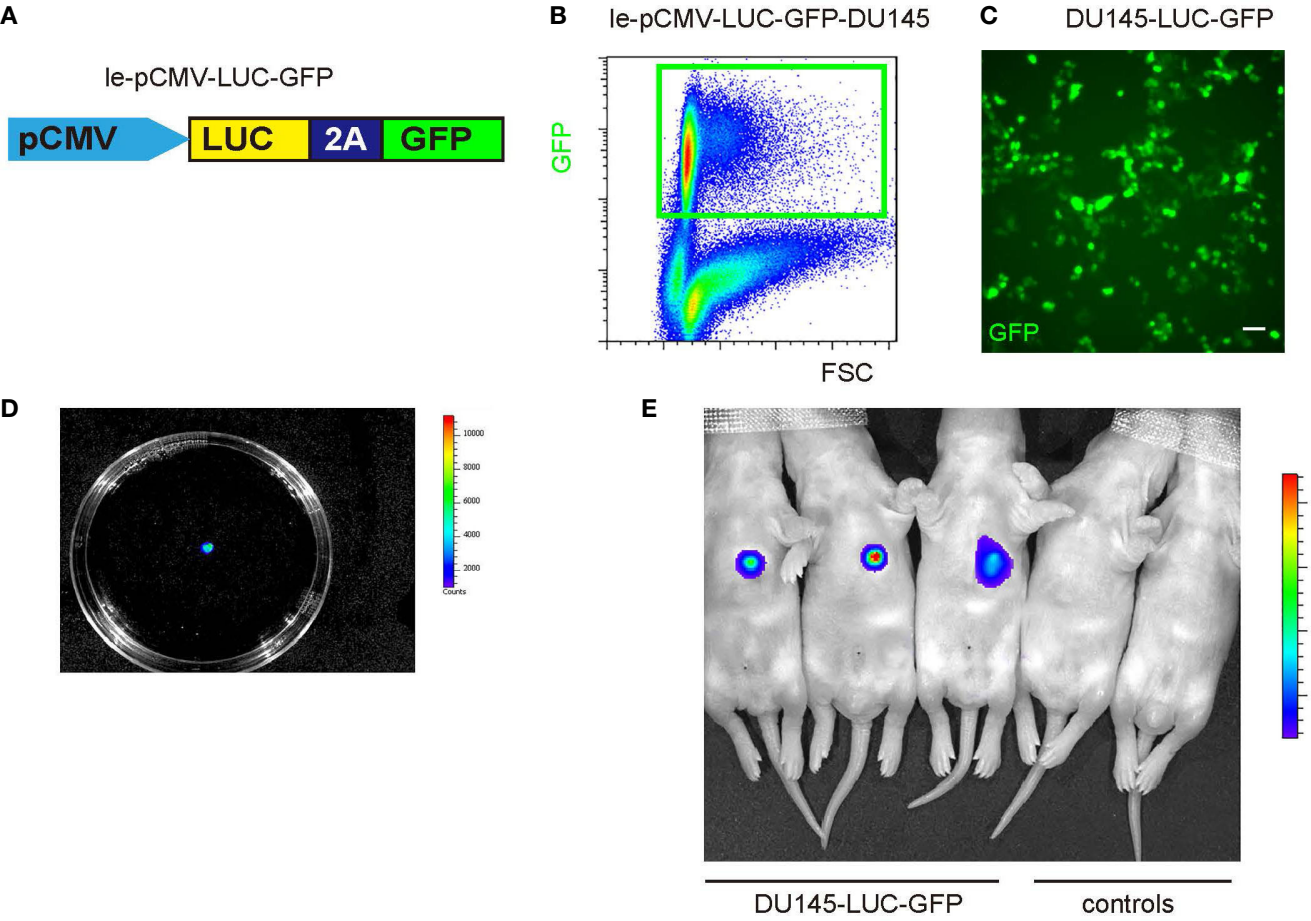
Establishment of a mouse PrC model **(A)** A diagram of a lenti vector carrying a luciferase and a green fluorescent protein (GFP) reporter under a CMV promoter. **(B)** The lenti-pCMV-LUC-GFP was used to transduce DU145 cells, sorted by flow cytometry as GFP-positive cells. **(C)** GFP-positive cells show green fluorescence. **(D)** The lenti-pCMV-LUC-GFP-transduced DU146 cells received luciferin to show the presence of luciferase expression in the cells. **(E)** LUC-GFP-DU145 cells were subcutaneously injected into nude mice to establish the PrC model and detected by bioluminescence (three left mice). Two control mice that did not receive tumor cell implantation were shown as controls (two right mice). Scale bar is 20 µm.

To dissect the potential molecular mechanisms underlying the crosstalk between TAM and PrC, we first used a lentivirus vector bearing both luciferase and GFP reporters under a CMV promoter to allow cell tracing and visualization of tumor cells (**Figure 2A**). A PrC cell line, DU145, which was genetically labeled with both GFP and luciferase, can be sorted with GFP expression by flow cytometry (**Figures 2B, C**) or traced with luciferase activity (**Figure 2D**). PrC was formed through subcutaneous grafting of the transduced DU145 cells to immune-deficient nude mice. PrC model establishment was proved by detection of luciferase activity at the transplanted location (**Figure 2E**).

### PrC with high PDGF expression and TAM with high PDGFR expression

**Figure 3:**
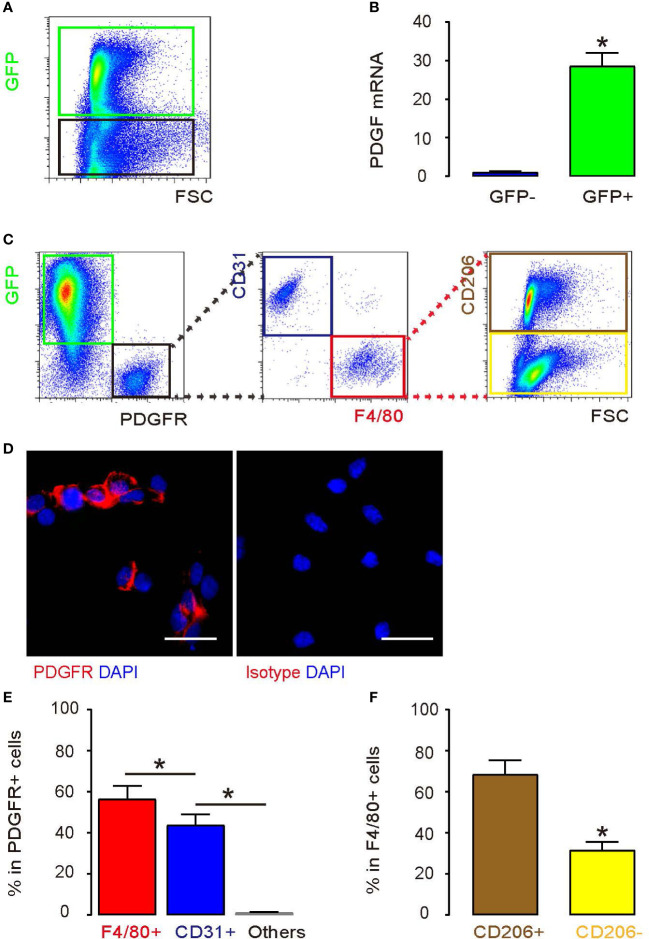
PrC cells express a high level of PDGF and TAMs express a high level of PDGFR **(A)** PrC cells from the mice model were detected by green fluorescence. **(B)** PDGF mRNA level of GFP-positive cells vs. GFP-negative cells. **(C)** Analyses of GFP-positive cells and PDGFR-positive cells from the PrC mouse model. PDGFR+ cells were divided into two populations based on CD31 and F4/80 expression. PDGFR+ F4/80+ cells were further analyzed for CD206 expression. The gating strategy for the flow cytometry was shown. **(D)** Staining with a PDGFR antibody or isotype control on green cells. **(E)** % of F4/80+, CD31+, and other cells in PDGFR+ cells measured by flow cytometry. **(F)** % of CD206+, CD206- cells in PDGFR+F4/80+ cells. *p < 0.05. *n* = 5. Scale bars are 20 µm.

PDGFR is the unique receptor for PDGF. Thus, we tested how PDGFR alpha (simplified as PDGFR throughout this article) and PDGF were expressed on PrC cells and the macrophages in the generated tumor in mice. The implanted tumor was digested into single cells for flow cytometry analysis. We found that most of the cells in the tumor expressed with GFP (**Figure 3A**). The implanted DU145 cells were green, while other cells, such as inflammatory cells, neurons, mesenchymal cells, endocrine cells in tumor block, were non-green. Next, the PDGF levels in GFP+ versus GFP- cell fractions were examined by using RT-qPCR analysis. According to the result, it was the green tumor cells rather than non-tumor cells that expressed high PDGF (**Figure 3B**). Later, PDGFR was detected exclusively in the non-green cell fraction by flow cytometry (**Figure 3C**), confirmed by immunocytochemistry for PDGFR on the non-green cell fraction (**Figure 3D**). CD31 (an endothelial cell marker) and F4/80 (a macrophage marker) were analyzed in PDGFR+ cells, and the results showed that the majority of the PDGFR+ cells were either F4/80+ cells or CD31+ cells (**Figures 3C, E**). Furthermore, more than 70% of the F4/80+ macrophages are CD206+, which represented an M2 or TAM phenotype (**Figures 3C, F**).

### Regulation of macrophage polarization from PrC cells substantializes PrC cell growth

**Figure 4:**
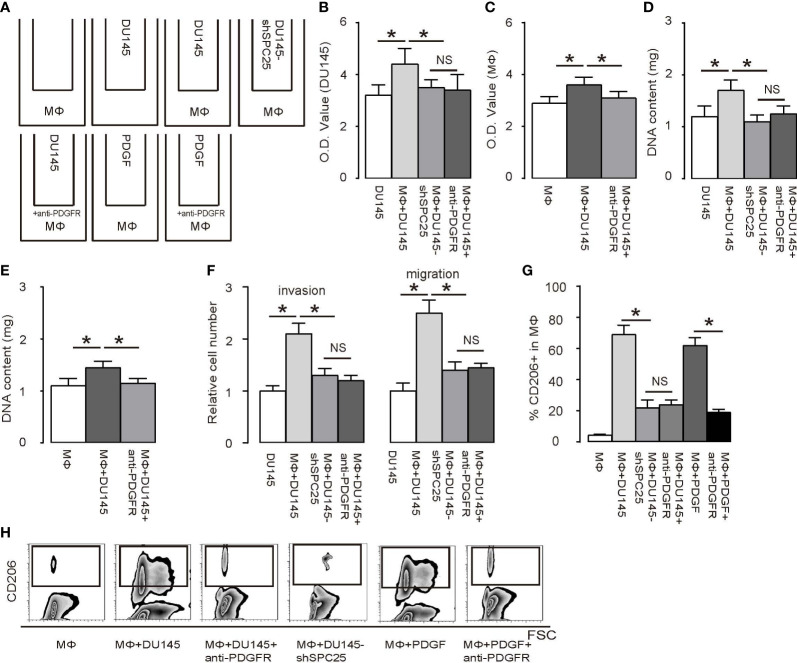
PrC cells regulate macrophage polarization, which, in turn, mediates PrC cell growth **(A)** A diagram of a transwell co-culture system for DU145 cells and bone-marrow-derived macrophages (MΦ). Group 1: macrophages alone; Group 2: DU145 cells alone; Group 3: co-culture of macrophages and DU145; Group 4: co-culture of macrophages and DU145-shSPC25; Group 5: co-culture of macrophages and DU145 with presence of an anti-PDGFR antibody; Group 6: macrophages alone with presence of PDGF; Group 7: macrophages alone with presence of PDGF and the anti-PDGFR antibody. Cells were incubated for 2 days. **(B, C)** An MTT assay was performed in DU145 cells **(B)** and in macrophages **(C)**. **(D, E)** DNA content determination on DU145 cells **(D)** and in macrophages **(E)**. **(F)** Cell invasion and migration in macrophages. **(G, H)** Flow cytometry analysis of CD206+ cells in macrophages, shown by quantification **(G)** and by representative flowcharts **(H)**. *p < 0.05. NS, non-significant. *n* = 5.

Next, a transwell co-culture experiment including DU145 with bone-marrow-derived macrophages was performed to dissect the crosstalk between PrC and macrophages. Group 1: macrophages alone; Group 2: DU145 cells alone; Group 3: co-culture of macrophages and DU145; Group 4: co-culture of macrophages and DU145-shSPC25; Group 5: co-culture of macrophages and DU145 with presence of an anti-PDGFR antibody; Group 6: macrophages alone with presence of PDGF; Group 7: macrophages alone with presence of PDGF and the anti-PDGFR antibody (**Figure 4A**). According to the results from an MTT assay, there was a remarkable increase in DU145 cell growth in 2 days, which was induced by macrophages. This increase in DU145 cell growth required PDGF/PDGFR signaling based on the fact that the influences of macrophages on increases of DU145 cells’ growth were abolished by either shSPC25 on DU145 cells or by the anti-PDGFR antibody (**Figure 4B**). PDGF by itself appeared to increase macrophage growth (**Figure 4C**). These data were confirmed by analysis of DNA content of the cells (**Figures 4D, E**). Moreover, the presence of macrophages significantly increased the invasiveness and migration of co-cultured DU145, which were abolished by either shSPC25 on DU145 cells or by the anti-PDGFR antibody (**Figure 4F**). Finally, increases in M2/TAM macrophages were induced by DU145 cells, which were abolished by either shSPC25 or by anti-PDGFR as well (**Figures 4G, H**). The influences of DU145 cells on the process of macrophage’s polarization were imitated by recombinant PDGF without need for the DU145 cells (**Figures 4G, H**). These results indicated that DU145 cells initiate macrophage polarization through SPC25/PDGF signaling, which is also a necessary process in DU145 cells’ growth.

### TGFβ1 secreted by polarized TAM promotes PrC cell growth

**Figure 5:**
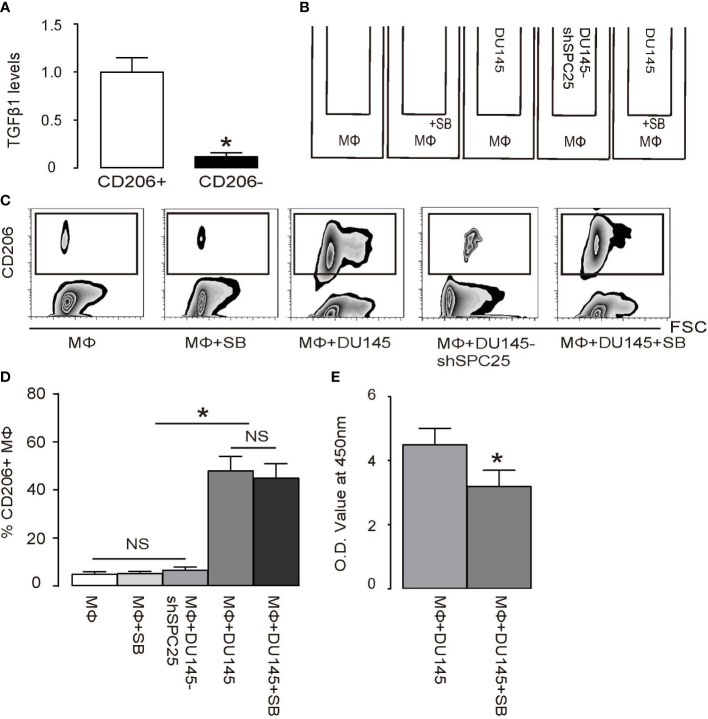
Polarized TAMs promote PrC cell growth through TGFβ1 **(A)** RT-qPCR for TGFβ1 mRNA level in CD206+ versus CD206- macrophages. **(B)** A transwell co-culture diagram of DU145 cells and bone-marrow-derived macrophages (MΦ). Group 1: MΦ. Group 2: MΦ with inhibitor SB431542 (SB). Group 3: MΦ and DU145 cells. Group 4: MΦ and DU145 cells transduced with shSPC25. Group 5: MΦ and DU145 cells with SB. Cells were incubated for 2 days. **(C, D)** Flow cytometry analysis of CD206+ cells in macrophages, shown by quantification **(C)** and by representative flowcharts **(D)**. **(E)** MTT assay on DU145 cells. *p < 0.05. NS, non-significant. *n* = 5.

To determine the molecular signals between polarized TAM and PrC cells, we focused on TGFβ1, which is a highly secreted factor by M2/TAM cells to exert a strong effect on tumor cell growth (20, 21). First, we found that the majority of TGFβ1 was derived from CD206+ M2/TAM but not from CD206- M1 macrophages (**Figure 5A**). SB431542 (SB), as an additional loss-of-functional control to suppress downstream TGFβ receptor signaling in the macrophage/DU145 cell co-culture system, is a specific inhibitor for TGFβ receptor I (22, 23). Macrophages alone, or macrophages with DU145-shSPC25 in the co-culture, were also applied as an additional control (**Figure 5B**). We found that the presence of SB to suppress TGFβ receptor signaling failed to alter macrophage polarization (**Figures 5C, D**), suggesting that TGFβ receptor signaling was not involved in the process of macrophage polarization triggered by PDGF. However, the presence of SB to inhibit TGFβ receptor signaling completely abolished the influences of macrophage polarization on the process of DU145 cells’ growth (**Figure 5E**).

### SPC25 activates PDGF through Egr-1

**Figure 6:**
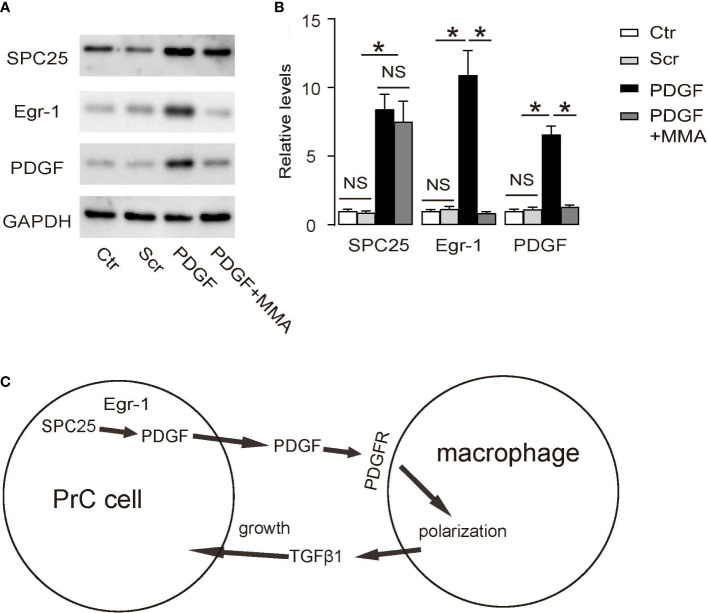
SPC25 activates PDGF through Egr-1 **(A, B)** Mithramycin A (MMA) was added at a dose of 0.1 µg/ml into the cultured LNCap cells. **(A)** Representative Western blots of SPC25, Egr-1, and PDGF, and **(B)** corresponding quantification. **(C)** A schematic of the model in this research: SPC25 in PrC cells enhances PDGF production likely through Egr-1 to polarize macrophages towards TAM through their surface expression of PDGFR. Polarized macrophages (TAM) produce TGFβ1 to promote growth of PrC cells. *p < 0.05. NS, non-significant. *n* = 5.

Lastly, we aimed to figure out how SPC25 activates PDGF. By searching public databases and previous publications, we found that SPC25 did not seem to directly activate PDGF transcription or directly interact with PDGF protein. Since Egr-1 has been shown to be a direct activator for PDGF (24), we used mithramycin A (MMA), an antibiotic that prevents binding of Egr1 to target promoters (25), in cultured PrC cells to determine whether the activation of PDGF may activate PDGF through Egr-1. Indeed, MMA-mediated suppression of Egr-1 abolished the activation of PDGF by SPC25, suggesting that SPC25 may activate PDGF through Egr-1 (**Figures 6A, B**). Together, our findings in this study suggest a molecular interaction between PrC and TAM through PDGF and TGFβ1, which were summarized in a schematic (**Figure 6C**).

## Discussion

Tumor growth is regulated by a complicated network that involves cancer cells, inflammatory cells, mesenchymal cells, endothelial cells, and other types of cells (26, 27). It was recently demonstrated that TAM plays a pivotal role in transducing signals in the process of tumor formation (28), but it is still uncertain how TAMs become activated in PrC. In this study, we focused on the effects of PrC-PDGF on TAM, likely through a paracrine regulatory pathway.

While it has been well-known that PDGF/PDGFR signaling is important for growth of tumor vessel, here we further showed that it coordinated the crosstalk between TAM and PrC in facilitating tumor growth. It was newly demonstrated by a report that M2 macrophages were infiltrated in the injured pancreas, and these M2 macrophages secreted high levels of TGFβ1, which enhanced beta cell replication (18). A similar process was also reported in cardiac muscle regeneration, in which macrophages were recruited into mesenchymal-stem-cell-transplanted injured heart to secrete BMP7 against the fibrogenic effect of TGFβ1 by macrophages, as well as to work as the catalyzer in enhancing angiogenesis and reviving cardiac muscle (19). We have proved that high TGFβ1 was secreted by M2 macrophages, which confirmed results in the two above-mentioned reports. Therefore, the trophic effects of M2 macrophages on cell growth appear to benefit tissue regeneration after injury but could be detrimental in tumor. The autocrine effects of PDGF have been proved to be important in some tumors (29). However, it seemed not critical in PrC, because little expression of PDGFR was detected in GFP+ tumor cells.

Since Egr-1 has been shown to be a direct activator for PDGF (24), we used MMA (25) in cultured PrC cells and found that MMA-mediated suppression of Egr-1 abolished the activation of PDGF by SPC25. Since both SPC25 and Egr-1 are primarily known as regulators of mitosis, it may be interesting to further study their interaction that regulates cell proliferation under physiological and pathological conditions.

Although here we did not study the crosstalk among TAM, PrC, and endothelial cells, previous studies have proved that TAMs possess significant influences on tumor vascular growth through production and release of a number of pro-angiogenetic factors. Moreover, PDGF has been demonstrated to promote mesenchymal stromal cell chemotaxis to the tumor microenvironment, in which it promotes both growth of tumor cells and tumor-associated endothelial cells. Hence, the PDGF/PDGFR signaling may play a central role in the regulation of tumor angiogenesis as well as tumor growth, through interaction of tumor cells, TAMs, and tumor endothelial cells. This question may be further addressed in future studies.

Here, we show the two stages interceded by PDGF/PDGFR signaling and TGFβ receptor signaling that coordinate the interaction between PrC and TAM. We also showed that the PrC cell growth was enhanced by the TAM, which had been polarized in a process that required TGFβ signaling. To the best of our knowledge, the involvement of this signaling pathway (SPC25/PDGF/TGFβ) in the crosstalk between PrC cells and TAM has not been reported before. Our study should provide novel insights into the intervention of PrC.

## Data availability statement

The original contributions presented in the study are included in the article/supplementary material. Further inquiries can be directed to the corresponding authors.

## Ethics statement

The studies involving human participants were reviewed and approved by Affiliated People’s Hospital of Jiangsu University. The patients/participants provided their written informed consent to participate in this study. The animal study was reviewed and approved by Affiliated People’s Hospital of Jiangsu University.

## Author contributions

The study conception and design were done by FC, JH, and YL. The collection and assembly of data were carried out by FC, ZX, JH, and YL. Data analysis and interpretation were performed by FC, JH, and YL. Manuscript was written by FC. All authors approved the manuscript to be published.

## Funding

This work was funded by the Social Development Plan of Jiangsu Province-Standardization of key disease diagnosis and treatment project (BE2016715), the Jiangsu Province Youth Medical Key Talent Program (QNRC2016457), the Natural Science Foundation of Jiangsu Province (BK20191221), and the Jiangsu Provincial Health Commission Project (H2017089).

## Conflict of interest

The authors declare that the research was conducted in the absence of any commercial or financial relationships that could be construed as a potential conflict of interest.

## Publisher’s note

All claims expressed in this article are solely those of the authors and do not necessarily represent those of their affiliated organizations, or those of the publisher, the editors and the reviewers. Any product that may be evaluated in this article, or claim that may be made by its manufacturer, is not guaranteed or endorsed by the publisher.
